# Decoding PANDAS with biocomputing: mapping purinergic dysregulation in neuroimmune crosstalk

**DOI:** 10.1097/MS9.0000000000004129

**Published:** 2025-10-17

**Authors:** Tanzeela Khan, Muhammad Talha, Mahnoor Fatima, Abdullah Imtiaz, Fahad Khan

**Affiliations:** a Department of Medicine, Dow Medical College, Dow University of Health Sciences, Karachi, Pakistan; b Department of Medicine, King Edward Medical University, Lahore, Pakistan; c Department of Medicine, Shaheed Ziaur Rahman Medical College, Rajshahi Medical University, Bogura, Bangladesh; d Department of Medicine, Wah Medical College, Wah Cantt, Pakistan

*To the Editor,*

Pediatric Autoimmune Neuropsychiatric Disorders Associated with Streptococcal infections or PANDAS is characterized by the sudden onset of neuropsychiatric symptoms, including obsessive compulsive disorder (OCD), tic disorders, and psychosis in children, following infection with group A beta-hemolytic Streptococcus pyogenes^[1]^. Emerging evidence suggests that the disruption of dopaminergic signaling underlies the behavioral and movement abnormalities. Studies demonstrate the role of autoantibodies as the key mediators in this dysregulation, targeting primarily D1 receptors, and in some cases, D2 receptors, particularly in children with choreatic movements. These receptor-mediated antibodies are considered potential biomarkers of disease activity, linking immune response to altered neurotransmission^[2]^. This is in line with the TITAN Guidelines on the need for transparency in AI use in healthcare^[3]^.

While dopaminergic alterations in PANDAS are well-established, the disruptions in the purinergic pathway remain largely unexplored. This connection has been suggested by the close postsynaptic interaction between adenosine receptor, or A2AR, and D2R, whereby dopaminergic disruption may induce secondary aberration in purine signaling^[4]^. Moreover, studies in Parkinson’s disease (PD) model demonstrate extracellular catabolism of synaptically released ATP into adenosine via CD73, which then activates A2AR, thus regulating corticostriatal plasticity. Notably, this study highlights the dual effect of A2AR across disease stages, where A2AR overfunctions in the pre-symptomatic phase, resulting in abnormally increased synaptic plasticity, followed by dampening of A2AR activity in the symptomatic phase. Other neurodegenerative models, like Alzheimer’s, show similar mechanisms, contributing to neuropsychiatric manifestations. These parallels raise the possibility that analogous aberration of A2AR in PANDAS could amplify immune-mediated dopaminergic alterations, culminating in OCD and psychotic symptoms^[5]^.

In order to investigate this, pathway analysis of purine metabolism and purinergic signaling genes, such as CD73, ADA, and the members of P2RX/P2RY receptor families, can be employed. Curated resources, such as Kyoto Encyclopedia of Genes and Genomes and Reactome, as well as metabolomics and transcriptomics tools, can be used to map perturbed nodes. This pathway mapping would provide a strong foundational basis in modeling how autoimmune triggers, such as antineuronal antibodies, interact with adenosine receptors and interfere with the purinergic pathway, leading to the potentiation of neuropsychiatric features. Furthermore, computational modeling could simulate the dynamics of dopaminergic-purinergic crosstalk under immune stress conditions, predicting timings and severity of neuropsychiatric flare following streptococcal infection. Such predictive modeling would represent a paradigm shift in clinical management, enabling identification of high-risk periods and opening pathways for anticipatory therapeutic interventions as well as tailored monitoring strategies for vulnerable patients^[6–8]^.

Continuing along this predictive model, there is also a need to investigate therapeutic frameworks that dive into the aberrant purinergic signaling. Parkinson asserts the possible effect of an A2AR antagonist in regulating neurotransmission by adenosine receptors that has proven effective in the correction of neuropsychiatric and cognitive impairments^[9]^. Since this has now been established as the role of A2AR antagonists in PD, they would be worth researching further in OCD and psychotic symptoms in PANDAS as well, where they may have a potentially transformative impact.

To conclude, by using biocomputing to analyze the purinergic pathways in PANDAS, we can understand how the immune response leads to an imbalance in the brain functions. This directly links molecular research to improved patient care. When combined with the treatment methods, this method promises a more complete and effective future for managing the disease.

## Data Availability

Not applicable to this study.

## Research registration unique identifying number (UIN)

No human subjects were involved.

## References

[1] Moretti G Pasquini M Mandarelli G. What every psychiatrist should know about PANDAS: a review. Clin Pract and Epidemiol Mental Health 2008;4:13. 10.1186/1745-0179-4-13 PMC2413218 18495013

[2] Menendez CM Zuccolo J Swedo SE. Dopamine receptor autoantibody signaling in infectious sequelae differentiates movement versus neuropsychiatric disorders. JCI Insight 2024;9:e164762. 39325550 10.1172/jci.insight.164762 PMC11601707

[3] Agha RA Mathew G Rashid R. Transparency in the reporting of Artificial INtelligence – the TITAN guideline. Prem J Sci 2025;10:100082.

[4] Prasad K de Vries EF Elsinga PH. Allosteric interactions between adenosine A2A and dopamine D2 receptors in heteromeric complexes: biochemical and pharmacological characteristics, and opportunities for PET imaging. Int J Mol Sci 2021;22:1719. 33572077 10.3390/ijms22041719 PMC7915359

[5] Gonçalves FQ Matheus FC Silva HB. Increased ATP release and higher impact of adenosine A2A receptors on corticostriatal plasticity in a rat model of presymptomatic Parkinson’s disease. Mol Neurobiol 2023;60:1659–74. 36547848 10.1007/s12035-022-03162-1 PMC9899190

[6] Huang Z Xie N Illes P. From purines to purinergic signalling: molecular functions and human diseases. Sig Transduct Target Ther 2021;6:162. 10.1038/s41392-021-00553-z PMC8079716 33907179

[7] Pasquini S Contri C Merighi S. Adenosine receptors in neuropsychiatric disorders: fine regulators of neurotransmission and potential therapeutic targets. Int J Mol Sci 2022;23:1219. 35163142 10.3390/ijms23031219 PMC8835915

[8] Swedo SE. Pediatric autoimmune neuropsychiatric disorders associated with streptococcal infections (PANDAS). Mol Psychiatry 2002;7:S24–5. 12142939 10.1038/sj.mp.4001170

[9] Uchida SI Kadowaki-Horita T Kanda T. Effects of the adenosine A2A receptor antagonist on cognitive dysfunction in Parkinson’s disease. Int Rev Neurobiol 2014;119:169–89. 25175966 10.1016/B978-0-12-801022-8.00008-8

